# The Association of Salt Intake and Non-alcoholic Fatty Liver Disease in People With Type 2 Diabetes: A Cross-Sectional Study

**DOI:** 10.3389/fnut.2022.943790

**Published:** 2022-07-13

**Authors:** Fuyuko Takahashi, Yoshitaka Hashimoto, Ayumi Kaji, Ryosuke Sakai, Yuka Kawate, Takuro Okamura, Noriyuki Kitagawa, Hiroshi Okada, Naoko Nakanishi, Saori Majima, Takafumi Osaka, Takafumi Senmaru, Emi Ushigome, Masahide Hamaguchi, Michiaki Fukui

**Affiliations:** ^1^Department of Endocrinology and Metabolism, Graduate School of Medical Science, Kyoto Prefectural University of Medicine, Kyoto, Japan; ^2^Department of Diabetes and Endocrinology, Matsushita Memorial Hospital, Moriguchi, Japan; ^3^Department of Diabetology, Kameoka Municipal Hospital, Kameoka, Japan

**Keywords:** salt intake, diet, NAFLD, type 2 diabetes, nutrition

## Abstract

**Objectives:**

Non-alcoholic fatty liver disease (NAFLD), which has a close relationship with type 2 diabetes (T2D), is related to salt intake in the general population. In contrast, the relationship between salt intake and the presence of NAFLD in patients with T2D has not been clarified.

**Methods:**

Salt intake (g/day) was assessed using urinary sodium excretion, and a high salt intake was defined as an intake greater than the median amount of 9.5 g/day. Hepatic steatosis index (HSI) ≥ 36 points was used to diagnosed NAFLD. Odds ratios of high salt intake to the presence of NAFLD were evaluated by logistic regression analysis.

**Results:**

The frequency of NAFLD was 36.5% in 310 patients with T2D (66.7 ± 10.7 years old and 148 men). The patients with high salt intake had a higher body mass index (25.0 ± 4.0 vs. 23.4 ± 3.8 kg/m^2^, *p* < 0.001) than those with low salt intake. HSI in patients with high salt intake was higher than that in patients with low salt intake (36.2 ± 6.2 vs. 34.3 ± 5.5 points, *p* = 0.005). In addition, the presence of NALFD in patients with high salt intake was higher than that in patients with low salt intake (44.5% vs. 28.4%, *p* = 0.005). High salt intake was associated with the prevalence of NAFLD [adjusted odds ratio, 1.76 (95% confidence interval: 1.02–3.03), *p* = 0.043].

**Conclusion:**

This cross-sectional study revealed that salt intake is related to the prevalence of NAFLD in patients with T2D.

## Introduction

Non–alcoholic fatty liver disease (NAFLD) is associated with chronic liver disease (1). NAFLD is regarded as the hepatic phenotype of ectopic accumulation of fat due to abdominal obesity and insulin resistance, and its existence relates to the accumulation of fat in visceral, perivascular, epicardial, and intramuscular tissues (2, 3). Patients with type 2 diabetes (T2D) often suffer from NAFLD (3). The presence of NAFLD is especially high in people with obesity and/or T2D, and it has

been found that NAFLD is present in approximately 50–60% of patients with T2D (4, 5). Preexisting diabetes was increasing the risk of NAFLD and death related to the liver in prospective studies (6, 7). Therefore, NAFLD in patients with T2D deserves more caution than in people without diabetes.

Lifestyle, including dietary habits, affects to NAFLD development. Excess energy intake increases hepatic fat mass (4), and eating fast has been related to the prevalence of NAFLD in patients with T2D (8). Moreover, an association between salt intake and NAFLD in the general population has been reported (9, 10). High salt intake raises blood pressure and increases the risk factor for cardiovascular disease (CVD) (11, 12). Furthermore, the relationship between high salt intake and a risk of obesity were shown in previous studies (13–17). High salt intake has been established as a risk of metabolic disorders (18–21). However, the association between salt intake and NAFLD in patients with T2D has not been clarified yet. Thus, this cross-sectional study intended to reveal the correlation between salt intake and NAFLD in patients with T2D.

## Materials and Methods

### Study Participants

Since 2014, we have been conducting a prospective cohort study, the KAMOGAWA-DM cohort study, which aims to identify the natural history of people with diabetes (22). In the present study, we enrolled outpatients of the Department of Endocrinology and Metabolism at Kyoto Prefectural University of Medicine (KPUM) Hospital (Kyoto, Japan) or the Department of Diabetology at Kameoka Municipal Hospital (Kameoka, Japan) who completed questionnaires between January 2016 and December 2018. This study excluded people with the following characteristics: non-T2D, absent data on hepatic steatosis index (HSI), viral hepatitis, liver cancer, alcohol consumption ≥ 20 g/day (23), absent data on urinary creatinine (Cr) and sodium (Na), and incomplete questionnaires. The approve of present study was obtained from the ethics committee of KPUM (No. RBMR-E-466-6) and this study was executed according to the Declaration of Helsinki. Written informed consent was provided from all participants.

### Data Collection

Smoking status and physical activity were assessed by questionnaires. “Habit of smoking” was defined as smoking cigarettes or another tobacco product currently. “Habit of exercise” was defined as an engagement in any type of exercise once a week or more. Additionally, all participants were questioned about the duration of their diabetes. Venous blood was gathered after fasting all night and checked for the following factors: plasma glucose, glycosylated hemoglobin (HbA1c), triglycerides, high-density lipoprotein cholesterol, gamma-glutamyl transpeptidase (γ-GTP), alanine aminotransferase (ALT), aspartate aminotransferase (AST), and creatinine (Cr). Estimated glomerular filtration rate (eGFR) was calculated as 194 × age^−0.287^ × Cr^−1.094^ (if women, ×0.739) (mL/min/1.73 m^2^) as defined by the Japanese Society of Nephrology (24).

Body mass index (BMI) was evaluated by dividing body weight (kg) by height^2^ (m^2^). Ideal body weight (IBW) was calculated as 22 × (square of the participant's height [m^2^]) (25). In addition, data on medications, including antidiabetic and antihypertensive drugs, were gathered from patients' records. Blood pressure was tested automatically using an HEM-906 device (OMRON, Kyoto, Japan) in a sitting posture after 5 min of rest in a quiet room. Hypertension was defined as the use of antihypertensive drugs, systolic blood pressure ≥ 140 mmHg, and/or diastolic pressure ≥ 90 mmHg (26).

### Questionnaire for Dietary Habit

We evaluated the participants' habitual food and nutrient intake during the previous 1 month using a brief-type self-administered diet history questionnaire (BDHQ). The validity and detail of the BDHQ have been shown previously (27). Energy intake (kcal/day), protein intake (g/day), carbohydrate intake (g/day), fat intake (g/day), alcohol consumption (g/day) and fiber intake (g/day) were assessed using the BDHQ. Furthermore, energy (kcal/IBW/day), protein (g/IBW/day), carbohydrate (g/IBW/day), fat (g/IBW/day) intakes were estimated by each intake divided by IBW.

### Definition of Salt Intake

The patients supplied samples of urine from their second early morning urination. Urinalysis was performed to assess densities of Cr (mg/dL) and Na (mg/dL). We estimated the 24 h salt intake with the Tanaka formula (28, 29): Estimated 24 h salt intake (g/day) = [21.98 × (urinary Na (mg/dL)/ urinay Cr/ 10 × (−2.043 × age + 14.89 × body weight (kg) + 16.14 × height (cm) −2244.5)^∧^0.392]/ 17.

Patients with high salt intake were defined as those consuming more than the median intake of 9.5 g/day.

### Definition of NAFLD

Patients who consumed alcohol were excluded from the study. HSI (30), which is calculated using the ALT/AST ratio, sex, BMI, and impaired fasting glucose (defined as blood glucose levels > 110 mg/dL) was utilized for the prevalence of fatty liver. The formula for HSI was as follows: HSI = 8 × (ALT/AST) + BMI [+2, if impaired fasting glucose (all of the participants in the present study); + 2, if women]. Patients having NAFLD were defined as those with a HSI score ≥ 36 points.

### Statistical Analysis

Data are shown as a mean ± standard deviation (SD) or frequencies of potential confounding variables. Patients were divided into two groups according to their salt intake. The differences in the categorical and continuous variables were assessed by the Student's *t*-test and chi-square test, respectively.

Logistic regression analyses were performed to investigate the association between having NAFLD and taking high salt intake. The independent variables were age, HbA1c, sodium glucose cotransporter-2 inhibitor, insulin treatment, glucagon-like peptide-1 receptor agonist, duration of diabetes, exercise, smoking, energy and dietary fiber intake.

Statistical significance was set at *p* < 0.05. Statistical analyses were performed using EZR (Saitama Medical Center, Jichi Medical University, Saitama, Japan) (31).

## Results

**Figure 1:**
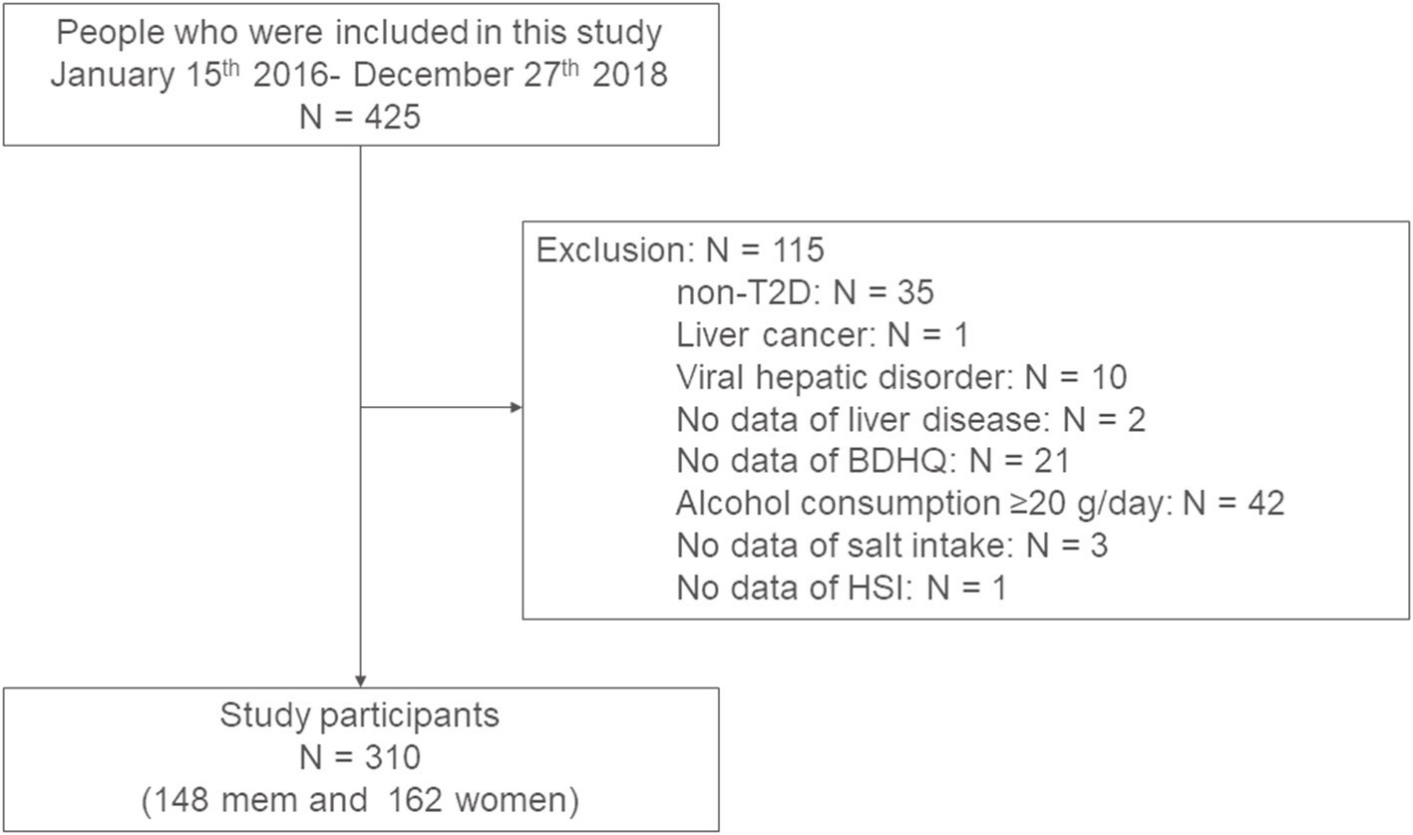
Study flow diagram for the registration of patients. BDHQ, a brief-type self-administered diet history questionnaire; HSI, hepatic steatosis index; T2D, type 2 diabetes.

The present study included 425 patients. We excluded 115 patients: 35 patients were not T2D, one patient had liver cancer, 10 patients had viral hepatitis, 2 patients had not known liver disease, 21 patients did not complete questionnaires, 42 patients had the habit of drinking alcohol, 3 patients did not have urinary data, and one patient did not calculate HSI due to lack of data; thus, the final study population consisted of 310 patients (148 men and 162 women) (Figure 1).

The characteristics of the study participants are indicated in Table 1. Mean age and BMI were 66.7 ± 10.7 years, and 24.2 ± 4.0 kg/m^2^, respectively. Mean HSI was 35.3 ± 5.9 points and the percentage of patients with NAFLD was 36.5%. In addition, the mean salt intake was 9.4 ± 2.5 g/day.

**Table 1:** Clinical characteristics of study participants.

	**All** ***N*** **= 310**
Age (years)	66.7 (10.7)
Sex (men/women)	148 [47.7%] / 162 [52.3%]
Duration of diabetes (years)	14.2 (10.0)
Family history of diabetes (-/+)	172 [55.5%] / 138 [44.5%]
Body mass index (kg/m^2^)	24.2 (4.0)
Systolic blood pressure (mmHg)	133.4 (18.3)
Diastolic blood pressure (mmHg)	78.4 (11.1)
Antihypertensive drugs (-/+)	148 [47.7%] / 162 [52.3%]
Presence of hypertension (-/+)	106 [34.2%] / 204 [65.8%]
Insulin (-/+)	238 [76.8%] / 72 [23.2%]
SGLT2 inhibitor (-/+)	259 [83.5%] / 51 [16.5%]
GLP-1 receptor agonist (-/+)	259 [83.5%] / 51 [16.5%]
Habit of smoking (-/+)	273 [88.1%] / 37 [11.9%]
Habit of exercise (-/+)	155 [50.0%] / 155 [50.0%]
HbA1c (mmol/mol)	56.5 (13.5)
HbA1c (%)	7.3 (1.2)
Creatinine (umol/L)	71.7 (30.7)
eGFR (ml/min/1.73 m^2^)	69.6 (19.1)
Triglycerides (mmol/L)	1.5 (0.8)
HDL cholesterol (mmol/L)	1.5 (0.4)
Aspartate aminotransferase (IU/L)	23.3 (14.5)
Alanine aminotransferase (IU/L)	22.8 (9.6)
Gamma-glutamyl transferase (IU/L)	32.7 (32.0)
HSI (point)	35.3 (5.9)
The presence of NAFLD (-/+)	197 [63.5%] / 113 [36.5%]
Urinary Creatinine (umol/L)	87.5 (56.3)
Urinary Na (umol/L)	104.0 (47.3)
Salt intake (g/day)	9.4 (2.5)
Total energy intake (kcal/day)	1674.4 (563.1)
Energy intake (kcal/IBW/day)	29.8 (10.0)
Total protein intake (g/day)	71.3 (27.9)
Protein intake (g/IBW/day)	1.3 (0.5)
Total fat intake (g/day)	54.3 (21.8)
Fat intake (g/IBW/day)	1.0 (0.4)
Total carbohydrate intake (g/day)	217.9 (82.0)
Carbohydrate intake (g/IBW/day)	3.9 (1.4)
Dietary fiber intake (g/day)	12.3 (4.9)
Carbohydrate-to-dietary fiber intake ratio	19.0 (6.8)

*Data were expressed as mean (standard deviation) or number [%]. eGFR, estimated glomerular filtration rate; GLP-1, glucagon-like peptide-1; HDL, high-density lipoprotein; HSI, hepatic steatosis index; SGLT2, sodium glucose cotransporter-2*.

The characteristics of the patients according to salt intake are indicated in Table 2. The patients with high salt intake were younger (64.9 ± 10.9 vs. 68.6 ± 10.2 years, *p* = 0.002) and had a higher BMI (25.0 ± 4.0 vs. 23.4 ± 3.8 kg/m^2^, *p* < 0.001) than those with low salt intake. HSI in patients with high salt intake was higher than that in patients with low salt intake (36.2 ± 6.2 vs. 34.3 ± 5.5 points, *p* = 0.005). The presence of NALFD in patients with high salt intake was higher than that in patients with low salt intake (44.5% vs. 28.4%, *p* = 0.005).

**Table 2:** Clinical characteristics according to salt intake.

	**Estimated salt intake (low)** ***N*** **= 155**	**Estimated salt intake (high)** ***N*** **= 155**	***P***
Age (years)	68.6 (10.2)	64.9 (10.9)	0.002
Sex (men/women)	73 [47.1%] / 82 [52.9%]	75 [48.4%] / 80 [51.6%]	0.909
Duration of diabetes (years)	15.0 (10.8)	13.4 (9.2)	0.159
Family history of diabetes (-/+)	80 [51.6%] / 75 [48.4%]	92 [59.4%] / 63 [40.6%]	0.209
Body mass index (kg/m^2^)	23.4 (3.8)	25.0 (4.0)	<0.001
Systolic blood pressure (mmHg)	133.7 (17.9)	133.2 (18.7)	0.814
Diastolic blood pressure (mmHg)	77.4 (11.6)	79.4 (10.6)	0.119
Antihypertensive drugs (-/+)	73 [47.1%] / 82 [52.9%]	75 [48.4%] / 80 [51.6%]	0.909
Presence of hypertension (-/+)	75 [48.4%] / 80 [51.6%]	74 [47.7%] / 81 [52.3%]	1.000
Insulin (-/+)	121 [78.1%] / 34 [21.9%]	117 [75.5%] / 38 [24.5%]	0.687
SGLT2 inhibitor (-/+)	135 [87.1%] / 20 [12.9%]	124 [80.0%] / 31 [20.0%]	0.126
GLP-1 receptor agonist (-/+)	134 [86.5%] / 21 [13.5%]	125 [80.6%] / 30 [19.4%]	0.220
Habit of smoking (-/+)	140 [90.3%] / 15 [9.7%]	133 [85.8%] / 22 [14.2%]	0.293
Habit of exercise (-/+)	82 [52.9%] / 73 [47.1%]	73 [47.1%] / 82 [52.9%]	0.363
HbA1c (mmol/mol)	7.3 (1.3)	7.3 (1.2)	0.766
HbA1c (%)	56.7 (13.7)	56.3 (13.4)	0.766
Creatinine (umol/L)	74.0 (36.3)	69.4 (23.9)	0.183
eGFR (mL/min/1.73 m^2^)	67.2 (18.1)	72.0 (19.9)	0.025
Triglycerides (mmol/L)	1.4 (0.9)	1.5 (0.8)	0.100
HDL cholesterol (mmol/L)	1.6 (0.4)	1.5 (0.4)	0.053
Aspartate aminotransferase (IU/L)	23.2 (10.4)	22.5 (8.7)	0.501
Alanine aminotransferase (IU/L)	23.2 (14.8)	23.4 (14.3)	0.894
Gamma-glutamyl transferase (IU/L)	33.7 (38.4)	31.7 (24.1)	0.587
HSI (point)	34.3 (5.5)	36.2 (6.2)	0.005
Presence of NAFLD (-/+)	111 [71.6%] / 44 [28.4%]	86 [55.5%] / 69 [44.5%]	0.005
Urinary Creatinine (umol/L)	114.2 (61.3)	60.7 (33.9)	<0.001
Urinary Na (umol/L)	87.5 (38.4)	120.6 (49.7)	<0.001
Salt intake (g/day)	7.4 (1.4)	11.3 (1.6)	<0.001
Total energy intake (kcal/day)	1,657.9 (563.0)	1,691.0 (564.6)	0.605
Energy intake (kcal/IBW/day)	29.9 (10.2)	29.7 (9.8)	0.877
Total protein intake (g/day)	72.3 (29.4)	70.3 (26.3)	0.531
Protein intake (g/IBW/day)	1.3 (0.6)	1.2 (0.5)	0.223
Total fat intake (g/day)	54.3 (24.0)	54.2 (19.6)	0.984
Fat intake (g/IBW/day)	1.0 (0.4)	1.0 (0.4)	0.593
Total carbohydrate intake (g/day)	212.4 (75.2)	223.4 (88.1)	0.236
Carbohydrate intake (g/IBW/day)	3.8 (1.3)	3.9 (1.5)	0.552
Dietary fiber intake (g/day)	12.3 (4.6)	12.3 (5.2)	0.892
Carbohydrate-to-dietary fiber intake ratio	18.6 (6.5)	19.3 (7.1)	0.337

*Data were expressed as mean (standard deviation) or number [%]. The difference between group was evaluated by Student's t-test or chi-square test. eGFR, estimated glomerular filtration rate; GLP-1, glucagon-like peptide-1; HDL, high-density lipoprotein; HSI, hepatic steatosis index; SGLT2, sodium glucose cotransporter-2*.

The association between salt intake and the prevalence of NAFLD is shown in Table 3. High salt intake was related to the presence of NAFLD [adjusted odds ratio, 1.76 (95% confidence interval: 1.02−3.03), *p* = 0.043].

**Table 3:** Odds ratio of salt intake on the presence of NAFLD.

	**Model1**		**Model 2**		**Model 3**	
	**OR (95% CI)**	***p***	**OR (95% CI)**	***p***	**OR (95% CI)**	***p***
Salt intake (high)	2.02 (1.26–3.24)	0.003	1.72 (1.01–2.94)	0.047	1.76 (1.02–3.03)	0.043
Age (years)	-	-	0.97 (0.94–0.99)	0.017	0.97 (0.94–0.995)	0.020
Duration of diabetes (years)	-	-	0.94 (0.91–0.97)	<0.001	0.94 (0.90–0.97)	<0.001
HbA1c (mmol/mol)	-	-	1.02 (0.997–1.04)	0.085	1.02 (0.997–1.04)	0.084
Insulin treatment	-	-	0.85 (0.43–1.68)	0.641	0.94 (0.47–1.88)	0.868
SGLT2 inhibitor	-	-	2.50 (1.25–4.99)	0.010	2.31 (1.14–4.70)	0.020
GLP-1 receptor agonist	-	-	3.17 (1.51–6.67)	0.003	2.88 (1.35–6.13)	0.006
Habit of exercise	-	-	-	-	1.14 (0.52–2.53)	0.742
Habit of smoking	-	-	-	-	1.14 (0.52–2.53)	0.742
Energy intake (kcal/IBW/day)	-	-	-	-	1.04 (1.00–1.07)	0.036
Dietary fiber intake (g/day)	-	-	-	-	0.91 (0.84–0.97)	0.008

*Model 1 is unadjusted; Model 2 is adjusted for age, duration of diabetes, HbA1c, insulin treatment, SGLT2 inhibitor, GLP-1 receptor agonist; Model 3 is adjusted for age, duration of diabetes, HbA1c, insulin treatment, SGLT2 inhibitor, GLP-1 receptor agonist, habit of exercise, habit of smoking, energy intake, dietary fiber intake*.

## Discussion

This is the initial study to clarify the relationship between salt intake and the prevalence of NAFLD in patients with T2D. The findings of the present study indicated that high salt intake is related to the presence of NAFLD in patients with T2D.

There are several possible interactions between high salt intake and NAFLD. NAFLD is regarded as the hepatic element of metabolic syndrome, with inordinate accumulation of fat and insulin resistance acting functioning as the major contributors in its pathophysiology (32, 33). High salt intake is known to induce insulin resistance (34) and white adipose tissue mass (13, 15, 35). The present study indicates that high salt intake might be associated with the presence of NAFLD because it promotes insulin resistance and fat accumulation. Previous studies have found that a high salt intake is associated with NAFLD in the general population (9, 10). Additionally, animal studies have revealed that high salt diet decreases the liver's antioxidant defenses (36) and may promote inflammation and fibrosis in liver (37). In a previous study, high salt intake led to an increase in the sodium levels in liver tissue whereas it was accompanied by a decline in mitochondrial respiratory activity (38). Mitochondrial dysfunction enhances hepatocyte steatosis (38). Moreover, recent experimental studies have shown that high salt intake induces leptin resistance *via* activating the aldose reductase–fructokinase pathway, production of endogenous fructose and increased white adipose tissue mass *via* leptin production and adipocyte hypertrophy in the liver (14, 35, 39). Leptin resistance leads to fatty fiver (14). Animal studies have revealed that leptin prevents ectopic fat accumulation and lipotoxicity (40). Leptin resistance might facilitate the accumulating fat in the liver and explain the occurrence of hepatic steatosis. The relationship between leptin resistance and NAFLD has been reported in patients with T2D (41).

This study had some limitations. First, salt intake was not accurately measured. Although the Na excretion, which was estimated using the aforementioned formula, has been reported to correlate with the measured 24 h Na excretion (*r* = 0.54, *p* < 0.01) (29), a previous study showed that data from the Tanaka formula are underestimated when excretion is low and are overestimated when excretion is high (42). Thus, to accurately assess salt intake, multiple sampling of 24 h urinary collections were necessary. Second, plasma leptin levels were not included. Therefore, the causal association between salt intake, leptin levels, and NAFLD is not clear. Third, the results of BDHQ reflect the previous month's dietary habits. Thus, it is possible that the results of BDHQ may not reflect the content of food intake when estimating the 24 h salt intake using the Tanaka formula. Fourth, due to the cross-sectional nature of this study, it was impossible to exhibit a causal association. We need further research, such as conducting a longitudinal study. Furthermore, previous studies have showed that people with high salt intake have unhealthy lifestyles, and that there is a higher risk of obese than people without high salt intake (43, 44). However, this study did not fully consider lifestyle. Therefore, there might be unknown confounders. Fifth, the impact of each antidiabetic or antihypertensive medication on sodium excretion is different (45–51). However, this study did not fully consider the impact of antidiabetic or antihypertensive medications on sodium excretion. Moreover, NAFLD itself may affect sodium excretion by affecting the renin-angiotensin system (52). Finally, diagnosis of NAFLD was not performed using liver biopsy, although it is the gold standard. Moreover, we did not use other non-invasive techniques, such as ultrasound, FibroScan, or MRI, for the diagnosis of fatty liver (53). However, the correlation between HSI and the definition of NAFLD using ultrasonography has been assessed (30).

In conclusion, to the best of our knowledge, the present study shows that salt intake is related to the prevalence of NAFLD in patients with T2D. It is suggested that the control of salt intake is crucial not only for blood pressure and CVD prevention but also for the prevention of NAFLD in patients with T2D.

## Data Availability Statement

The raw data supporting the conclusions of this article will be made available by the authors, without undue reservation.

## Ethics Statement

The studies involving human participants were reviewed and approved by Kyoto Prefectural University of Medicine. The patients/participants provided their written informed consent to participate in this study.

## Author Contributions

FT designed the work, analyzed and interpreted the data, and wrote the manuscript. YH conceived and designed the work, acquired, analyzed, interpreted the data, and revised the manuscript. AK and RS conceived and designed the study, acquired data, and contributed to the discussion. YK, TOk, NK, TOs, NN, SM, TS, HO, and EU acquired data and contributed to the discussion. MH designed the work, acquired the data, and discussed the results. MF conceived and designed the study, acquired and interpreted the data, and revised the manuscript. All authors have read and agreed to the published version of the manuscript, contributed to the article, and approved the submitted version.

## Conflict of Interest

YH received personal fees from Novo Nordisk Pharma Ltd., Mitsubishi Tanabe Pharma Corp., Kowa Company Ltd., Sanofi K.K., Takeda Pharmaceutical Co., Ltd., Ono Pharmaceutical Co., Ltd., Daiichi Sankyo Co., Ltd., and Sumitomo Dainippon Pharma Co., Ltd. outside of the submitted work. HO received grant support from the Japan Society for the Promotion of Science and personal fees from Daiichi Sankyo Co., Ltd., Takeda Pharmaceutical Co., Ltd., Sumitomo Dainippon Pharma Co., Ltd., Novo Nordisk Pharma Ltd., MSD K.K., Kyowa Hakko Kirin Company Ltd., Kowa Pharmaceutical Co., Ltd., Eli Lilly Japan K.K., Ono Pharmaceutical Co., Ltd., Kissei Pharmaceutical Co., Ltd., Sanofi K.K., and Mitsubishi Tanabe Pharma Corporation. NN received grant support from the Japan Society for the Promotion of Science (JSPS KAKENHI grant numbers: 19K23999 and 20K16158) and the Japan Food Chemical Research Foundation, and personal fees from Novo Nordisk Pharma Ltd. and Kowa Pharmaceutical Co., Ltd. TOs received grants from Combi Corporation and personal fees from Toa Eiyo Corp., Mitsubishi Tanabe Pharma Corp., Daiichi Sankyo Co., Ltd., Novo Nordisk Pharma Ltd., Nippon Boehringer Ingelheim Co., Ltd., Ono Pharmaceutical Co., Ltd., Kyowa Kirin Co., Ltd., Sumitomo Dainippon Pharma Co., Ltd., MSD K.K., Takeda Pharmaceutical Co., Ltd., Kowa Pharma Co., Ltd., Eli Lilly Japan K.K., and AstraZeneca K.K., outside of the submitted work. TS received personal fees from Kyowa Hakko Kirin Co., Ltd., Astellas Pharma Inc., Mitsubishi Tanabe Pharma Co., Kowa Pharma Co., Ltd., Sanofi K.K., Taisho Toyama Pharma Co., Ltd., Kissei Pharma Co., Ltd., MSD K.K., Novo Nordisk Pharma Ltd., Ono Pharma Co., Ltd., Eli Lilly Japan K.K., and Takeda Pharma Co., Ltd., outside the submitted work. EU received grant support from the Japanese Study Group for Physiology and Management of Blood Pressure, Astellas Foundation for Research on Metabolic Disorders (grant number: 4024), Japan Society for the Promotion of Science, Mishima Kaiun Memorial Foundation, and personal fees from Sumitomo Dainippon Pharma Co., Ltd., Mitsubishi Tanabe Pharma Corporation, Nippon Boehringer Ingelheim Co., Ltd., Sanofi K.K., Kowa Pharmaceutical Co., Ltd., Daiichi Sankyo Co., Ltd., Kyowa Hakko Kirin Co., Ltd., AstraZeneca K.K., Novo Nordisk Pharma Ltd., Ono Pharmaceutical Co., Ltd., Taisho Pharmaceutical Co., Ltd., Takeda Pharmaceutical Company Ltd., and MSD K.K. outside of the submitted work. The donated fund laboratory of diabetes therapeutics is an endowment department supported by an unrestricted grant from Taiyo Kagaku Co., Ltd., Taisho Pharmaceutical Co., Ltd., and Ono Pharmaceutical Co., Ltd. MH received grants from Yamada Bee Farm, Oishi Kenko Inc., Nippon Boehringer Ingelheim Co., Ltd., AstraZeneca K.K., and Ono Pharma Co., Ltd. and personal fees from Eli Lilly, Japan, Sanofi K.K., Sumitomo Dainippon Pharma Co., Ltd., Daiichi Sankyo Co., Ltd., Mitsubishi Tanabe Pharma Corp., AstraZeneca K.K., Ono Pharma Co., Ltd., and Kowa Pharma Co., Ltd., outside the submitted work. MF received grants from Eli Lilly, Japan, K.K., Nippon Boehringer Ingelheim Co., Ltd., Sanwa Kagagu Kenkyusho Co., Ltd., Oishi Kenko Inc., MSD K.K., Kowa Pharma Co., Ltd., Kissei Pharma Co., Ltd., Sumitomo Dainippon Pharma Co., Ltd., Ono Pharma Co. Ltd., Mitsubishi Tanabe Pharma Corp., Abbott Japan Co., Ltd., Daiichi Sankyo Co., Ltd., Johnson & Johnson K.K. Medical Co., Astellas Pharma Inc., Kyowa Kirin Co., Ltd., Novo Nordisk Pharma Ltd., Yamada Bee Farm, Taisho Pharma Co., Ltd., Terumo Corp., Takeda Pharma Co., Ltd., Tejin Pharma Ltd., Sanofi K.K., Nippon Chemiphar Co., Ltd., and TERUMO CORPORATION, and personal fees from Astellas Pharma Inc., Nippon Boehringer Ingelheim Co., Ltd., Sanwa Kagaku Kenkyusho Co., Ltd., MSD K.K., Mochida Pharma Co., Ltd., Eli Lilly Japan K.K., Kissei Pharma Co., Ltd., AstraZeneca K.K., Mitsubishi Tanabe Pharma Corp., TERUMO CORPORATION, Daiichi Sankyo Co., Ltd., Bayer Yakuhin, Ltd., Takeda Pharma Co., Ltd., Teijin Pharma Ltd., Ono Pharma Co., Ltd., Taisho Pharma Co., Ltd., Kyowa Kirin Co., Ltd., Abbott Japan Co., Ltd., Sumitomo Dainippon Pharma Co., Ltd., Arkray Inc., Medtronic Japan Co., Ltd., Novo Nordisk Pharma Ltd., Kowa Pharma Co., Ltd., Nipro Corp., and Sanofi K.K., outside of the submitted work. The remaining authors declare that the research was conducted in the absence of any commercial or financial relationships that could be construed as a potential conflict of interest.

## Publisher's Note

All claims expressed in this article are solely those of the authors and do not necessarily represent those of their affiliated organizations, or those of the publisher, the editors and the reviewers. Any product that may be evaluated in this article, or claim that may be made by its manufacturer, is not guaranteed or endorsed by the publisher.

## References

[1] Younossi ZM Koenig AB Abdelatif D Fazel Y Henry L Wymer M. Global epidemiology of nonalcoholic fatty liver disease—Meta-analytic assessment of prevalence, incidence, and outcomes. Hepatology. (2016) 64:73–84. 10.1002/hep.28431 26707365

[2] Tilg H Moschen AR Roden M. NAFLD and diabetes mellitus. Nat Rev Gastroenterol Hepatol. (2017) 14:32–42. 10.1038/nrgastro.2016.147 27729660

[3] Adams LA Anstee QM Tilg H Targher G. Non-alcoholic fatty liver disease and its relationship with cardiovascular disease and other extrahepatic diseases. Gut. (2017) 66:1138–53. 10.1136/gutjnl-2017-313884 28314735

[4] Chalasani N Younossi Z Lavine JE Diehl AM Brunt EM Cusi K. The diagnosis and management of non-alcoholic fatty liver disease: Practice Guideline by the american association for the study of liver diseases, american college of gastroenterology, and the american gastroenterological association. Hepatology. (2012) 55:2005–23. 10.1002/hep.25762 22488764

[5] Dai W Ye L Liu A Wen SW Deng J Wu X. Prevalence of nonalcoholic fatty liver disease in patients with type 2 diabetes mellitus: a meta-analysis. Medicine. (2017) 96:e8179. 10.1097/MD.0000000000008179 28953675 PMC5626318

[6] Younossi ZM Gramlich T Matteoni CA Boparai N McCullough AJ. Nonalcoholic fatty liver disease in patients with type 2 diabetes. Clin Gastroenterol Hepatol. (2004) 2:262–5. 10.1016/S1542-3565(04)00014-X 15017611

[7] Porepa L Ray JG Sanchez-Romeu P Booth GL. Newly diagnosed diabetes mellitus as a risk factor for serious liver disease. CMAJ. (2010) 182:526–31. 10.1503/cmaj.092144 20566726 PMC2917963

[8] Takahashi F Hashimoto Y Kawano R Kaji A Sakai R Kawate Y. Eating fast is associated with nonalcoholic fatty liver disease in men but not in women with type 2 diabetes: a cross-sectional study. Nutrients. (2020) 12:1–12. 10.3390/nu12082174 32707957 PMC7468737

[9] Shen X Jin C Wu Y Zhang Y Wang X Huang W. Prospective study of perceived dietary salt intake and the risk of non-alcoholic fatty liver disease. J Hum Nutr Diet. (2019) 32:802–9. 10.1111/jhn.12674 31209928

[10] Huh JH Lee KJ Lim JS Lee MY Park HJ Kim MY. High dietary sodium intake assessed by estimated 24-h urinary sodium excretion is associated with NAFLD and hepatic fibrosis. PLoS ONE. (2015) 10:1–11. 10.1371/journal.pone.0143222 26571018 PMC4646649

[11] He FJ Li J MacGregor GA. Effect of longer term modest salt reduction on blood pressure: Cochrane systematic review and meta-analysis of randomised trials. BMJ. (2013) 346:f1325. 10.1136/bmj.f1136 23558162

[12] Strazzullo P D'Elia L Kandala NB Cappuccio FP. Salt intake, stroke, and cardiovascular disease: Meta-analysis of prospective studies. BMJ. (2009) 339:1296. 10.1136/bmj.b4567 19934192 PMC2782060

[13] Yoon YS Oh SW. Sodium density and obesity; the Korea national health and nutrition examination survey 2007-2010. Eur J Clin Nutr. (2013) 67:141–6. 10.1038/ejcn.2012.204 23249877

[14] Lanaspa MA Kuwabara M Andres-Hernando A Li N Cicerchi C Jensen T. High salt intake causes leptin resistance and obesity in mice by stimulating endogenous fructose production and metabolism. Proc Natl Acad Sci U S A. (2018) 115:3138–43. 10.1073/pnas.1713837115 29507217 PMC5866545

[15] Zhou L Stamler J Chan Q van Horn L Daviglus ML Dyer AR. Salt intake and prevalence of overweight/obesity in Japan, China, the United Kingdom, and the United States: the INTERMAP study. Am J Clin Nutr. (2019) 110:34–40. 10.1093/ajcn/nqz067 31111867 PMC6599742

[16] Larsen SC Ängquist L Sørensen TIA Heitmann BL. 24h urinary sodium excretion and subsequent change in weight, waist circumference and body composition. PLoS ONE. (2013) 8:e69689. 10.1371/journal.pone.0069689 23936079 PMC3723894

[17] Yoshida Y Kosaki K Sugasawa T Matsui M. High salt diet impacts the risk of sarcopenia associated with reduction of skeletal muscle. Nutrients. (2020) 1–14. 10.3390/nu12113474 33198295 PMC7696631

[18] Zivkovic AM German JB Sanyal AJ. Comparative review of diets for the metabolic syndrome: implications for nonalcoholic fatty liver disease. Am J Clin Nutr. (2007) 86:285–300. 10.1093/ajcn/86.2.285 17684197

[19] Yi SS Kansagra SM. Associations of sodium intake with obesity, body mass index, waist circumference, and weight. Am J Prev Med. (2014) 46:e53–5. 10.1016/j.amepre.2014.02.005 24842744

[20] Baudrand R Campino C Carvajal CA Olivieri O Guidi G Faccini G. Increased urinary glucocorticoid metabolites are associated with metabolic syndrome, hypoadiponectinemia, insulin resistance and β cell dysfunction. Steroids. (2011) 76:1575–81. 10.1016/j.steroids.2011.09.010 21996535

[21] Bibbins-Domingo K Chertow GM Coxson PG Moran AE Lightwood JM Pletcher MJ. Reductions in cardiovascular disease projected from modest reductions in dietary salt. N Eng J Med. (2010) 362:590–9. 10.1056/NEJMoa0907355 20089957 PMC3066566

[22] Sakai R Hashimoto Y Ushigome E Miki A Okamura T Matsugasumi M. Late-night-dinner is associated with poor glycemic control in people with type 2 diabetes: the KAMOGAWA-DM cohort study. Endocr J. (2018) 65:395–402. 10.1507/endocrj.EJ17-0414 29375081

[23] Kaji A Hashimoto Y Sakai R Okada H Hamaguchi M Ushigome E. Frequent usage of convenience stores is associated with low diet quality. Nutrients. (2019) 11:1–11. 10.3390/nu11061212 31141979 PMC6627471

[24] Matsuo S Imai E Horio M Yasuda Y Tomita K Nitta K. Revised equations for estimated GFR from serum creatinine in Japan. Am J Kidney Dis. (2009) 53:982–92. 10.1053/j.ajkd.2008.12.034 19339088

[25] Lemmens HJM Brodsky JB Bernstein DP. Estimating ideal body weight—A new formula. Obes Surg. (2005) 15:1082–3. 10.1381/0960892054621350 16105412

[26] Carretero OA Oparil S. Essential hypertension part I: definition and etiology. Circulation. (2000) 101:329–335. 10.1161/01.cir.101.3.329 10645931

[27] Kobayashi S Honda S Murakami K Sasaki S Okubo H Hirota N. Both comprehensive and brief self-administered diet history questionnaires satisfactorily rank nutrient intakes in Japanese adults. J Epidemiol. (2012) 22:151–9. 10.2188/jea.JE20110075 22343326 PMC3798594

[28] Koo HS Kim YC Ahn SY Oh SW Kim S Chin HJ. Estimating 24-hour urine sodium level with spot urine sodium and creatinine. J Korean Med Sci. (2014) 29:S97–102. 10.3346/jkms.2014.29.S2.S97 25317024 PMC4194291

[29] Tanaka T Okamura T Miura K Kadowaki T Ueshima H Nakagawa H. A simple method to estimate populational 24-h urinary sodium and potassium. J Hum Hypertens. (2002) 97–103. 10.1038/sj.jhh.1001307 11850766

[30] Lee JH Kim D Kim HJ Lee CH Yang JI Kim W. Journal of Korean Medical S*cience al. Hepatic steatosis index: a simple screening tool reflecting nonalcoholic fatty liver disease*. Dig Liver Dis. (2010) 42:503–8. 10.1016/j.dld.2009.08.002 19766548

[31] Kanda Y. Investigation of the freely available easy-to-use software “EZR” for medical statistics. Bone Marrow Transplant. (2013) 48:452–8. 10.1038/bmt.2012.244 23208313 PMC3590441

[32] Park JH. Insulin resistance in non-alcoholic fatty liver disease. Korean J Hepatol. (2006) 12:16–30. 16565603

[33] Koda M Kawakami M Murawaki Y Senda M. The impact of visceral fat in nonalcoholic fatty liver disease: cross-sectional and longitudinal studies. J Gastroenterol. (2007) 42:897–903. 10.1007/s00535-007-2107-z 18008034

[34] Ogihara T Asano T Ando K Sakoda H Anai M Shojima N. High-salt diet enhances insulin signaling and induces insulin resistance in Dahl salt-sensitive rats. Hypertension. (2002) 40:83–9. 10.1161/01.HYP.0000022880.45113.C9 12105143

[35] Fonseca-Alaniz MH Brito LC Borges-Silva CN Takada J Andreotti S Lima FB. High dietary sodium intake increases white adipose tissue mass and plasma leptin in rats. Obesity. (2007) 15:2200–8. 10.1038/oby.2007.261 17890487

[36] Dornas WC de Lima WG dos Santos RC da Costa Guerra JF de Souza MO Silva M. High dietary salt decreases antioxidant defenses in the liver of fructose-fed insulin-resistant rats. J Nutr Biochem. (2013) 24:2016–22. 10.1016/j.jnutbio.2013.06.006 24135554

[37] Uetake Y Ikeda H Irie R Tejima K Matsui H Ogura S. High-salt in addition to high-fat diet may enhance inflammation and fibrosis in liver steatosis induced by oxidative stress and dyslipidemia in mice. Lipids Health Dis. (2015) 14:2–9. 10.1186/s12944-015-0002-9 25888871 PMC4337194

[38] Gao P You M Li L Zhang Q Fang X Wei X. Salt-induced hepatic inflammatory memory contributes to cardiovascular damage through epigenetic modulation of SIRT3. Circulation. (2022) 145:375–91. 10.1161/CIRCULATIONAHA.121.055600 35100024

[39] Fonseca-Alaniz MH Takada J Andreotti S de Campos TBF Campãa AB Borges-Silva CN. High sodium intake enhances insulin-stimulated glucose uptake in rat epididymal adipose tissue. Obesity. (2008) 16:1186–92. 10.1038/oby.2008.69 18369340

[40] Lee Y Wang MY Kakuma T Wang ZW Babcock E McCorkle K. Liporegulation in diet-induced obesity: the antisteatotic role of hyperleptinemia. J Biol Chem. (2001) 276:5629–35. 10.1074/jbc.M008553200 11096093

[41] Cernea S Roiban AL Both E Hutanu A. Serum leptin and leptin resistance correlations with NAFLD in patients with type 2 diabetes. Diabetes Metab Res Rev. (2018) 34:e3050. 10.1002/dmrr.3050 30052309

[42] Dougher CE Rifkin DE Anderson CAM Smits G Persky MS Block GA. Spot urine sodium measurements do not accurately estimate dietary sodium intake in chronic kidney disease. Am J Clin Nutr. (2016) 104:298–305. 10.3945/ajcn.115.127423 27357090 PMC4962156

[43] Colin-Ramirez E McAlister FA Woo E Wong N Ezekowitz JA. Association between self-reported adherence to a low-sodium diet and dietary habits related to sodium intake in heart failure patients. J Cardiovasc Nurs. (2015) 30:58–65. 10.1097/JCN.0000000000000124 24598553

[44] Choi KH Park MS Kim JA Lim JA. Associations between excessive sodium intake and smoking and alcohol intake among Korean men: KNHANES V. Int J Environ Res Public Health. (2015) 12:15540–9. 10.3390/ijerph121215001 26670236 PMC4690937

[45] Ferrannini E Baldi S Frascerra S Astiarraga B Barsotti E Clerico A. Renal handling of ketones in response to sodium-glucose cotransporter 2 inhibition in patients with type 2 diabetes. Diabetes Care. (2017) 40:771–6. 10.2337/dc16-2724 28325783

[46] Moreno C Mistry M Roman RJ. Renal effects of glucagon-like peptide in rats. Eur J Pharmacol. (2002) 434:163–7. 10.1016/S0014-2999(01)01542-4 11779579

[47] Gupta AK Clark RV Kirchner KA. Effects of insulin on renal sodium excretion. Hypertension. (1992) 19:I78–82. 10.1161/01.hyp.19.1_Suppl.i78 1730458

[48] Endo Y Suzuki M Yamada H Horita S Kunimi M Yamazaki O. Thiazolidinediones enhance sodium-coupled bicarbonate absorption from renal proximal tubules via PPARγ-dependent nongenomic signaling. Cell Metab. (2011) 13:550–61. 10.1016/j.cmet.2011.02.015 21531337

[49] Packer M. Worsening heart failure during the use of DPP-4 inhibitors: pathophysiological mechanisms, clinical risks, and potential influence of concomitant antidiabetic medications. JACC Hear Fail. (2018) 6:445–51. 10.1016/j.jchf.2017.12.016 29525332

[50] Lim PO MacFadyen RJ Struthers AD. Is there a role for renin profiling in selecting chronic heart failure patients for ACE inhibitor treatment? Heart. (2000) 83:257–61. 10.1136/heart.83.3.257 10677399 PMC1729351

[51] Filipowicz E Staszków M. Diuretics. Wiad Lek. (2013) 66:319–23. 24490487

[52] Paschos P Tziomalos K. Nonalcoholic fatty liver disease and the renin-angiotensin system: Implications for treatment. World J Hepatol. (2012) 4:327–31. 10.4254/wjh.v4.i12.327 23355909 PMC3554795

[53] Ballestri S Nascimbeni F Baldelli E Marrazzo A Romagnoli D Targher G. Ultrasonographic fatty liver indicator detects mild steatosis and correlates with metabolic/histological parameters in various liver diseases. Metabolism. (2017) 72:57–65. 10.1016/j.metabol.2017.04.003 28641784

